# Evaluation of a strategy for enrolling the families of critically ill patients in research using limited human resources

**DOI:** 10.1371/journal.pone.0177741

**Published:** 2017-05-25

**Authors:** Alison E. Turnbull, Mohamed D. Hashem, Anahita Rabiee, An To, Caroline M. Chessare, Dale M. Needham

**Affiliations:** 1 Outcomes After Critical Illness and Surgery Group, Johns Hopkins University, Baltimore, Maryland, United States of America; 2 Division of Pulmonary & Critical Care Medicine, School of Medicine, Johns Hopkins University, Baltimore, Maryland, United States of America; 3 Department of Epidemiology, Bloomberg School of Public Health, Johns Hopkins University, Baltimore, Maryland, United States of America; 4 Cleveland Clinic, Department of Medicine, Cleveland, Ohio, United States of America; 5 Department of Internal Medicine, Yale School of Medicine, Yale University, New Haven, Connecticut, United States of America; 6 Department of Physical Medicine and Rehabilitation, School of Medicine, Johns Hopkins University, Baltimore, Maryland, United States of America; King Abdullah International Medical Research Center, SAUDI ARABIA

## Abstract

**Rationale:**

Clinical trials of interventions aimed at the families of intensive care unit (ICU) patients have proliferated but recruitment for these trials can be challenging.

**Objectives:**

To evaluate a strategy for recruiting families of patients currently being treated in an ICU using limited human resources and time-varying daily screening over 7 consecutive days

**Methods:**

We screened the Johns Hopkins Hospital medical ICU census 7 days per week to identify eligible family members. We then made daily, in-person attempts to enroll eligible families during a time-varying 2-hour enrollment period until families declined participation, consented, or were no longer eligible.

**Measurements and main results:**

The primary outcome was the proportion of eligible patients for whom ≥1 family member was enrolled. Secondary outcomes included enrollment of legal healthcare proxies, the consent rate among families approached for enrollment, and success rates for recruiting at different times during the day and week. Among 284 eligible patients, 108 (38%, 95% CI 32%-44%) had ≥1 family member enrolled, and 75 (26%, 95% CI 21%-32%) had their legal healthcare proxy enrolled. Among 117 family members asked to participate, 108 (92%, 95% CI 86%-96%) were enrolled. Patients with versus without an enrolled proxy were more likely to be white (44% vs. 30%, P = .02), live in a zip code with a median income of ≥$100,000 (15% vs. 5%, P = .01), be mechanically ventilated (63% vs. 47%, P = .01), die in the ICU (19% vs. 9%, P = .03), and to have longer ICU stays (median 5.0 vs. 1.8 days, P<.001). Day of the week and time of day were not associated with family presence in the ICU or consent rate.

**Conclusions:**

Family members were recruited for more than one third of eligible patients, and >90% of approached consented to participate. There are important demographic differences between patients with vs without an enrolled family member.

## Introduction

The majority of ICU patients lack decision-making capacity and rely on family members to communicate with clinicians.[1,2] In the past 15 years, the Society of Critical Care Medicine and American Thoracic Society have endorsed clinical practice guidelines and policies on supporting and engaging family members in shared decision making.[3,4] Research on ICU families has also proliferated over the past two decades. Investigators have explored the experiences of ICU families via qualitative interviews,[5–7] quantified conflict between families and clinicians,[8–10] assessed family understanding of prognostic information,[11–13] and trialed interventions to assist families making treatment decisions.[14,15] Recent research has demonstrated that many family members experience long-term mental health issues, including symptoms of depression[16–19] and post-traumatic stress disorder,[20–23] which have been termed post-intensive care syndrome-family or PICS-F.[24,25]

Recruiting families of ICU patients to participate in research can be challenging. Families who are caring for children, continuing employment, or traveling long distances to the hospital may visit infrequently or outside regular business hours. A recent study of bereaved ICU family members was unable to contact 69% of eligible family members because of inaccuracies in the patient record.[26] Families who are emotionally overwhelmed or distrustful may avoid the ICU or family meetings. Importantly, when the association between exposure and outcome differs between families who participate and those who do not, selection bias occurs.[27] These issues apply both to randomized trials and observational studies, and recruitment strategies for studies of ICU families must plan for these challenges.

Hence the objective of our research was to design and evaluate a strategy for recruiting family members of ICU patients exploiting a time-varying approach to daily screening within the common constraint in clinical research of having limited human resources. We evaluate the strategy’s ability to recruit a family member within 7 days of patient eligibility, as well as whether participants were the patient’s legal healthcare proxy, and the representativeness of recruited families. Moreover, we compare recruitment rates and family characteristics across different times and days of the week.

## Methods

### Setting

This evaluation was conducted prospectively as part of an ongoing pilot study to evaluate the acceptability of a brief educational intervention to prepare families to act as surrogate decision-makers in the medical ICU of Johns Hopkins Hospital in Baltimore, Maryland. The pilot study’s total budget of $5,000 restricted our ability to compensate study personnel. This subsequently limited the time available for recruiting and interviewing participants each week. All participants provided in-person, oral consent. Johns Hopkins Medicine IRB Committee X approved this study number: IRB00080137. The IRB deemed oral consent as appropriate because participating in the study did not involve any procedure for which written consent is normally required outside of the research context. Details of the intent of the proposed study, its design, the type of information collected, and potential risks and benefits of participation were discussed. The time, date, and identify of the participant was recorded in the study record.

### Participants

Study participants were the family members of all eligible patients. Patients became eligible 24 hours after medical ICU admission and remained eligible for 7 consecutive days or until death or ICU discharge. We waited 24 hours before approaching families to ensure they had an opportunity to speak to a clinician before enrollment. Patients who were incarcerated or <18 years old were not eligible. Family members were excluded from participation if they were unable to understand or speak English, <18 years old, or never visited the medical ICU. We defined family using the Society of Critical Care Medicine 2004–2005 task force definition which includes individuals who may be related or unrelated and with whom the patient has a significant relationship.[28]

### Procedure

Enrollment started on January 4^th^, 2016. We screened Johns Hopkins Hospital’s medical ICU census 7 days per week to identify eligible patients. Every day, a study team member was physically present in the medical ICU for a 2-hour period termed the “daily enrollment period.” The three team members trained to recruit and enroll family members were white females of similar age (25–35 years old). Each week, 2-hour enrollment periods were scheduled between 09:00 and 17:00 on three weekdays, between 17:00 and 21:00 on two weekdays, and between 09:00 and 17:00 on weekends. All communications with study participants were conducted in-person.

A family’s “recruitment window” started 24 hours after the patient’s admission to the medical ICU and continued for 7 consecutive days or until the patient’s death or ICU discharge. S1 Fig illustrates how recruitment using this strategy could have occurred for a single hypothetical patient. Permission to approach family members for consent was obtained from both the attending physician and bedside nurse. The legal healthcare proxies, defined as the patient’s healthcare agent(s), surrogate(s), or guardian under Maryland state law, was preferentially enrolled if more than one family member was present during a specific recruitment window.[29,30] If the family member initially enrolled was not the patient’s legal health care proxy using a standardized algorithm provided in S2 Fig, additional attempts were made to also enroll the legal healthcare proxy, so that a maximum of two family members were enrolled for each eligible patient. When a patient’s legal healthcare proxy did not wish to participate or was not expected to visit the ICU, another adult family member advocating for the patient was approached for enrollment.

We made daily attempts, 7 days per week, to enroll a family member for each eligible patient during the 7 day recruitment window until all available family members declined participation, a proxy consented, the patient was discharged from the medical ICU or the patient died. Availability of family during the daily recruitment period was recorded each day as one of the following (1) no family present, (2) no permission from attending physician or nurse, (3) family deferred participation, (4) family declined participation, or (5) family consented. Interviews with enrolled family members were conducted in a private area outside the patient’s room. Participants were asked to provide basic demographic information and feedback on the educational materials. Most interviews required between 15 and 30 minutes. Participants received a $10 gift card upon interview completion.

Data collection for all eligible patients included age, sex, race, zip code, location prior to hospitalization, admission diagnosis, advance directives at ICU admission, code status 24 hours after ICU admission, and presence of a phone number for an emergency contact in the electronic medical record. Length of hospital and ICU stay, disposition at hospital discharge, mechanical ventilation status (ever/never), and withdrawal of life support were collected at hospital discharge. We reviewed nursing records and consulted with the patient’s multidisciplinary clinical team to determine whether a patient had ever been visited by a family member during the recruitment window. Data collected about enrolled family members included relation to the patient, age, gender, race, years of formal education, and previous experience supporting a family member while in the ICU were obtained via in-person interview.

### Analysis

Our primary outcome was the proportion of eligible patients for whom at least 1 family member was enrolled. Family members were considered enrolled if they consented to participate and completed >50% of the survey. Secondary outcomes included the proportion of eligible patients for whom a legal healthcare proxy was enrolled, and the consent rate for families who were asked to participate. To estimate the proportion of eligible patients for whom at least 1 family member can be enrolled with a 5% margin of error and a 95% confidence level (α = .05), we planned to screen 369 consecutive ICU patients. For patients who were readmitted to the ICU during the study, only data from their first ICU admission was included in analysis.

To assess whether family members were more likely to be enrolled after 5pm on weekdays or on weekends versus during regular business hours, we conducted a patient-day level analysis. Patients contributed patient-days to this analysis during the recruitment window until death, ICU discharge, or enrollment of a legal proxy. In addition, we compared the characteristics of proxies and patients enrolled between 09:00 and 17:00 on weekdays versus outside of this time frame. Differences in continuous variables were tested using the Wilcoxon-Mann-Whitney two-sample test. All descriptive statistics and plots were generated using the R programming language version 3.3.0 (Vienna, Austria) using two-sided significance tests, with P<.05 indicating statistical significance.

## Results

**Fig 1:**
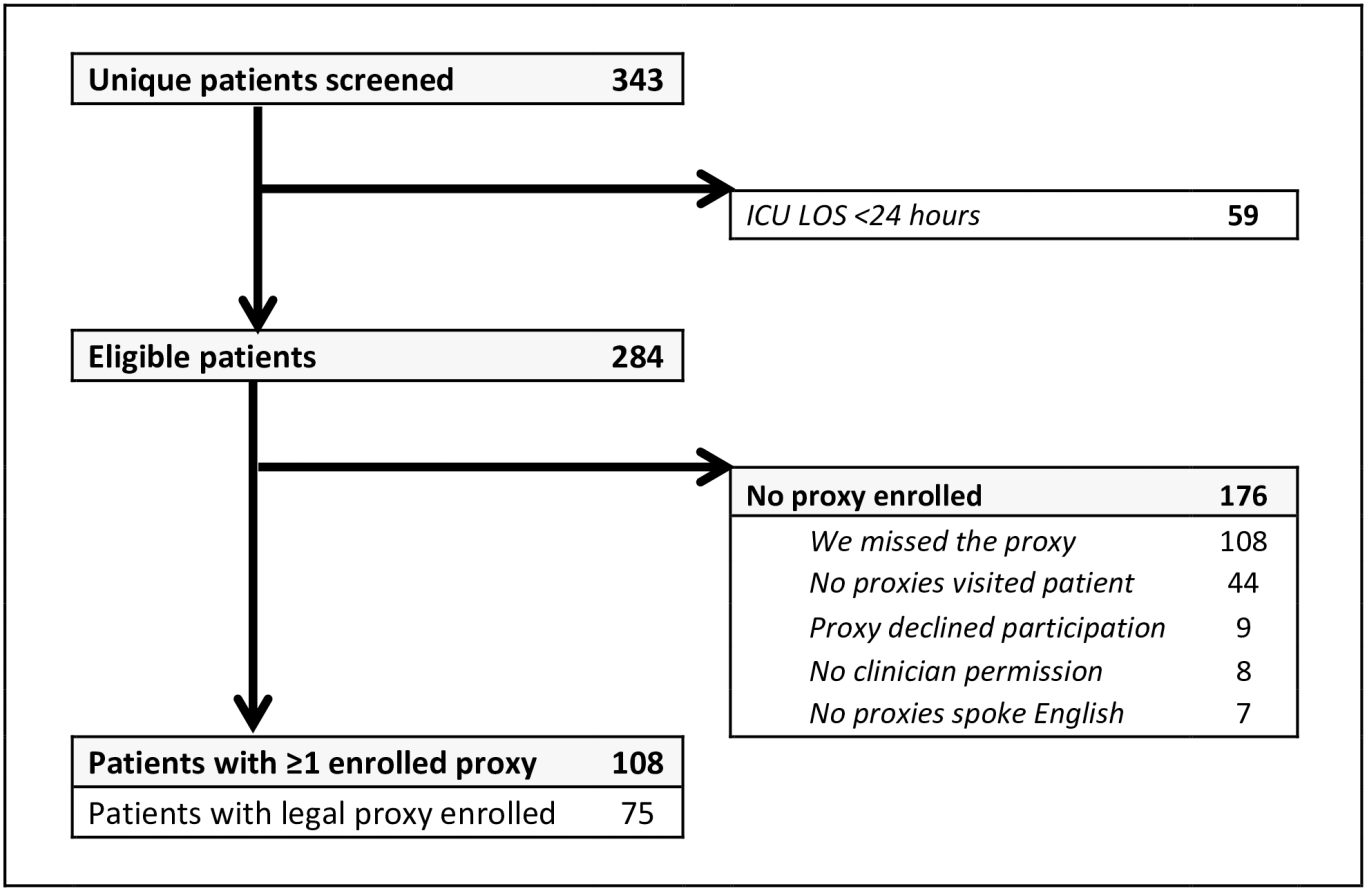
Patient screening, study eligibility and enrollment. 369 ICU admissions for 343 unique patients were screened. Only a patient's 1st ICU admission during the study period was analyzed. Eight patients had two proxies enrolled.

Over 96 days, there were 369 admissions to the medical ICU for 343 unique patients (Fig 1). There were 59 (17%) patients discharged within 24 hours of ICU admission, leaving the families of 284 patients eligible for enrollment. A total of 108 (38%, 95% CI 32% – 44%) eligible patients had at least one family member enrolled. We enrolled a legal healthcare proxy for 75 (26%, 95% CI 21% – 32%) eligible patients. Family members of 30 patients (28%, 95% CI 20% – 37%) reported that the patient had a document naming a legal healthcare agent, but only 5 (17%) of these patients had a copy of the document in their medical chart (S3 Fig).

Among 117 family members who were asked to participate, 108 (92%, 95% CI 86% – 96%) enrolled in the study. Eligible patients with versus without an enrolled proxy were more likely to be white (44% vs. 30%, P = .02), to live in a zip code with a median income of ≥$100,000 (15% vs. 5%, P = .01), to be mechanically ventilated (63% vs. 47%, P = .01), to die in the ICU (19% vs. 9%, P = .03) (Table 1) and to have longer ICU stays (median 5.0 vs. 2.5 days, P<.001).

**Table 1:** Characteristics of eligible patients by enrollment status.

	Enrolled^a^	Missed family	Proxies ineligible^b^	MD or RN Refused	Family Declined	Total
	(N = 108)	(N = 108)	(N = 51)	(N = 8)	(N = 9)	(N = 284)
**Age**, Median (IQR)	58 (48,69)	56 (45,68)	52 (44,62)	62 (23,66)	55 (51,69)	56 (45,67)
**Sex** N (%)						
Male	53 (49%)	54 (50%)	26 (51%)	1 (12%)	5 (56%)	139 (49%)
**Race** N (%)						
Black or African American	47 (44%)	59 (55%)	26 (51%)	5 (62%)	4 (44%)	141 (50%)
White	47 (44%)	35 (32%)	12 (24%)	1 (12%)	4 (44%)	99 (35%)
Other	14 (13%)	14 (13%)	13 (25%)	2 (25%)	1 (11%)	44 (15%)
**Location prior to hospitalization** N (%)						
House/Apt (independent)	71 (66%)	78 (72%)	36 (71%)	4 (50%)	8 (89%)	197 (69%)
House/Apt (with assistance)	28 (26%)	14 (13%)	8 (16%)	3 (38%)	1 (11%)	54 (19%)
Other	8 (7%)	13 (12%)	6 (12%)	1 (12%)	0 (0%)	28 (10%)
Unknown or missing	1 (1%)	3 (3%)	1 (2%)	0 (0%)	0 (0%)	5 (2%)
**Median income of zip code** N (%)^c^						
<$40K	31 (29%)	42 (39%)	16 (31%)	1 (12%)	3 (33%)	93 (33%)
$40K-$69K	40 (37%)	42 (39%)	25 (49%)	3 (38%)	1 (11%)	111 (39%)
$70K-$99K	20 (19%)	18 (17%)	7 (14%)	4 (50%)	4 (44%)	53 (19%)
≥$100K	16 (15%)	6 (6%)	2 (4%)	0 (0%)	1 (11%)	25 (9%)
**In chart at admission screening** N (%)						
Advance directive or POA listed in EMR	6 (6%)	5 (5%)	3 (6%)	1 (12%)	0 (0%)	15 (5%)
MOLST Form	5 (5%)	7 (6%)	2 (4%)	0 (0%)	0 (0%)	14 (5%)
**Code Status after 24 hours in ICU** N (%)						
Full code	94 (87%)	92 (85%)	47 (92%)	5 (62%)	9 (100%)	247 (87%)
Full code with treatment limitations	6 (6%)	8 (7%)	2 (4%)	2 (25%)	0 (0%)	18 (6%)
DNR and DNI	8 (7%)	8 (7%)	2 (4%)	1 (12%)	0 (0%)	19 (7%)
**Phone numbers for patient contacts recorded in EMR** N (%) *****						
Spouse / Girlfriend / Boyfriend	51 (47%)	26 (24%)	9 (18%)	0 (0%)	5 (56%)	91 (32%)
Adult child	36 (33%)	44 (41%)	13 (25%)	2 (25%)	2 (22%)	97 (34%)
Parent	19 (18%)	24 (22%)	7 (14%)	3 (38%)	1 (11%)	54 (19%)
Sibling	16 (15%)	14 (13%)	8 (16%)	0 (0%)	1 (11%)	39 (14%)
Other	10 (9%)	15 (14%)	8 (16%)	0 (0%)	0 (0%)	33 (12%)
Unknown or not reported	16 (15%)	22 (20%)	16 (31%)	4 (50%)	0 (0%)	58 (20%)
**Admission diagnosis**, N (%)						
Respiratory failure	47 (44%)	49 (45%)	16 (31%)	4 (50%)	2 (22%)	118 (42%)
Sepsis	23 (21%)	16 (15%)	3 (6%)	2 (25%)	0 (0%)	44 (15%)
Gastrointestinal	10 (9%)	13 (12%)	12 (24%)	0 (0%)	1 (11%)	36 (13%)
Cardiovascular	6 (6%)	11 (10%)	5 (10%)	0 (0%)	2 (22%)	24 (8%)
Other	22 (20%)	19 (18%)	15 (29%)	2 (25%)	4 (44%)	62 (22%)
**Mechanical ventilation, N (%)**						
Ever mechanically ventilated	68 (63%)	55 (51%)	17 (33%)	4 (50%)	7 (78%)	150 (53%)
**ICU Length of stay and disposition**						
ICU length of stay, median (IQR)	5 (3,9)	2 (2,4)	3 (2,4)	3 (2,3)	6 (4,7)	3 (1, 5)
Died in ICU, N (%)	20 (19%)	12 (11%)	0 (0%)	4 (50%)	0 (0%)	36 (13%)
Withdrawal of life-support, N (%)	21 (19%)	12 (11%)	0 (0%)	4 (50%)	0 (0%)	37 (13%)
**Hospital Length of stay and disposition**,						
Hospital length of stay, median (IQR)	11 (7,24)	10 (5,17)	6 (4,14)	6 (2,8)	15 (10,54)	9 (5, 18)
House/Apt (independent)	33 (31%)	52 (48%)	32 (63%)	0 (0%)	4 (44%)	121 (43%)
House/Apt (with home care)	14 (13%)	7 (6%)	5 (10%)	2 (25%)	1 (11%)	29 (10%)
Died	29 (27%)	17 (16%)	1 (2%)	4 (50%)	0 (0%)	51 (18%)
Hospice	8 (7%)	6 (6%)	0 (0%)	0 (0%)	0 (0%)	14 (5%)
Other including residential care	24 (22%)	26 (24%)	13 (25%)	2 (25%)	4 (44%)	69 (24%)

**Abbreviation**: DNI, Do not intubate; DNR, Do not resuscitate; EMR, Electronic medical record; ICU, Intensive care unit; IQR, Interquartile Range; MOLST, Maryland Medical Orders for Life Sustaining Treatment; POA, Power of Attorney.

^**a**^ Patients for whom any proxy was enrolled although 31% were not the patient’s legal healthcare proxy.

^**b**^ Patients with ineligible proxies included 44 patients for whom no proxy visited the ICU, and 7 patients for whom no proxy spoke English.

^**c**^ US Census Bureau 2010–2014; $41,819 median household income for Baltimore City; $74,194 median household income for MD state. No zip code was provided for 1 patient from Saudi Arabia with an enrolled proxy and 1 homeless patient with ineligible proxies.

Family members were equally likely to be present in the medical ICU on weekday evenings (30%), weekends (31%), and during daytime (09:00–17:00) on weekdays (29%) (Table 2). Similarly, there was little difference in agreement to participate by eligible family members approached during these 3 time frames (76%, 73% and 69%, respectively). Family members approached on Day 2 versus Day 1 were less likely to participate (80% vs 58%, P = .03); however, the consent rate rose on Days 3 and 4 (78% and 63%, respectively, Table 2).

**Table 2:** Proxy availability and enrollment.

Enrollment Day Characteristics	Patient-days		Eligible proxy^a^ present N (%)	P-value	Eligible proxies enrolled N (%)	P-value
**Timing of 2-hour daily enrollment period**						
Monday-Friday 09:00–17:00	224	64 (29%)		Ref	44 (69%)	Ref
Monday-Friday 17:00–21:00	152	46 (30%)		.81	35 (76%)	.53
Weekends 09:00–17:00	128	40 (31%)		.68	29 (73%)	.85
**Day of patient eligibility**						
1	229	80 (35%)		Ref	64 (80%)	Ref
2	118	36 (31%)		.48	21 (58%)	.03
3	70	18 (26%)		.20	14 (78%)	.76
4	37	8 (22%)		.16	5 (63%)	.36
5–7	50	8 (16%)		.01	4 (50%)	.07

^**a**^The following patients were excluded from day-level analysis: Patients without English-speaking family members, patients with ICU length of stay >24 hours who were discharged before the first 2-hour enrollment period, patients for whom clinician permission to approach family members was not obtained, and two potentially eligible patients who were mistakenly not added to the daily enrollment list on their 1^st^ day of eligibility. If the first proxy enrolled for a patient was not the legal healthcare proxy, subsequent days spent attempting to locate the legal proxy were excluded from day-level analysis.

**Fig 2:**
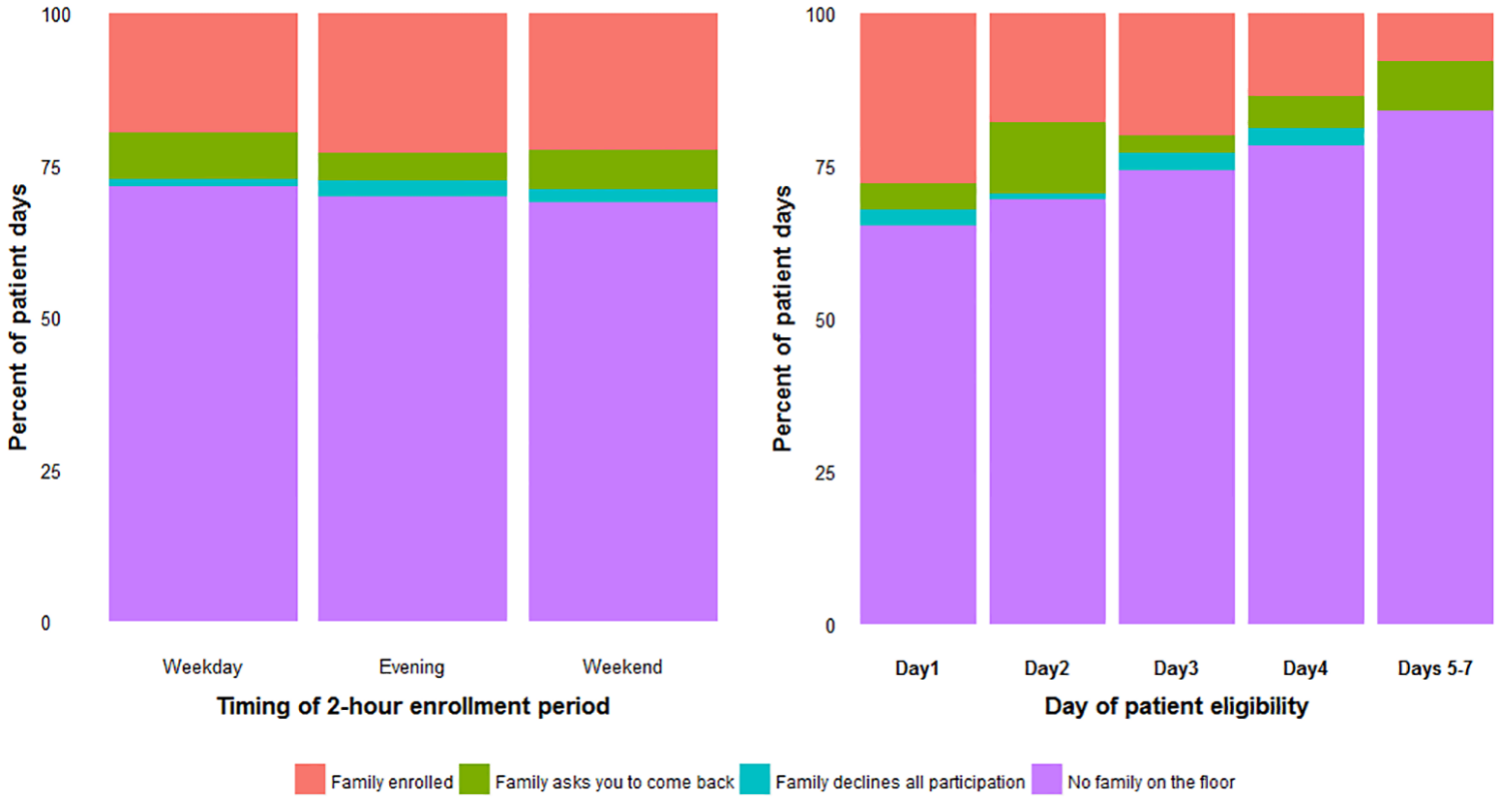
Patient-day level analysis of family presence and enrollment in the medical intensive care unit. The following patients were excluded from day-level analysis: Patients without English-speaking family members, patients with ICU length of stay >24 hours who were discharged before the first 2-hour enrollment period, patients for whom clinician permission to approach family members was not obtained, and two potentially eligible patients who were mistakenly not added to the daily enrollment list on their 1^st^ day of eligibility. If the first proxy enrolled for a patient was not the legal healthcare proxy, subsequent days spent attempting to locate the legal proxy were excluded from day-level analysis.

During the 7-day recruitment window, a family member was most likely to be enrolled on Day 1, with families less likely to be available in the ICU with each subsequent day of eligibility (Fig 2).

Among enrolled proxies, the median age was 51 years old, 74 (69%) were female, 61 (56%) were non-white, and 70 (65%) had prior experience supporting a loved one in an ICU (Table 3). There were no significant differences in the characteristics of proxies enrolled during weekdays versus evenings or weekends.

**Table 3:** Characteristics of enrolled proxies and their loved ones by enrollment time.

	Weekdays 9am– 5pm	Evenings & weekends	P-value
**Characteristics of Enrolled Proxies (N = 116)**	**(N = 47)**	**(N = 69)**	
**Age**^a^, Median (range)	50 (25, 70)	51 (18, 77)	.77
**Sex**^a^, N (%)			
Male	13 (28%)	21 (30%)	1.0
**Relationship to patient**, N (%)^d^			
Daughter	13 (28%)	16 (23%)	.43
Wife/Girlfriend	10 (21%)	19 (28%)	
Husband/Boyfriend	3 (6%)	11 (16%)	
Son	5 (11%)	3 (4%)	
Other	15 (32%)	19 (28%)	
**Race**^a^, N (%)			
Black or African American	21 (45%)	32 (46%)	.76
White	20 (43%)	29 (42%)	
Other	2 (4%)	6 (9%)	
**Years of Education**, Median (range)	14 (10, 21)	14 (10, 24)	.17
**Have you ever supported a loved one in ICU before?** N (%)^b^			
Yes	27 (57%)	43 (62%)	.64
No	17 (36%)	24 (35%)	
Unsure	3 (6%)	2 (3%)	
**Is the enrolled subject the patient's legal proxy?** N (%)			
Yes	29 (62%)	46 (67%)	.65
**Characteristics of Patients (N = 108)**	**(N = 44)**	**(N = 64)**	
**Median income of zip code**, N (%)^c^			
<$40K	11 (25%)	20 (31%)	.29
$40K-$69K	20 (45%)	20 (31%)	
$70K-$99K	8 (18%)	12 (19%)	
≥$100K	4 (9%)	12 (19%)	
**Length of stay**			
ICU length of stay, median (range)	5 (1, 26)	5 (2, 49)	.44
Hospital length of stay, median (range)	9 (2, 54)	12 (3, 218)	.05

**Abbreviations**: DNI, Do Not Intubate; DNR, Do Not Resuscitate; ICU, Intensive Care Unit; IQR, Interquartile Range

^**a**^ Proxies declined to report information on their age (n = 4), sex (n = 2), and race (n = 6).

^**b**^ Percentages do not sum to 100% due to rounding.

^**c**^ No zip code was provided for 1 patient from Saudi Arabia with a proxy enrolled on a weekday. US Census Bureau 2010–2014; $41,819 median household income for Baltimore City; $74,194 median household income for MD state.

^**d**^ We need to figure out how the 2 missing people were related

## Discussion

In this pilot study of a brief educational intervention for adult family members of patients in a racially and socio-economically diverse medical ICU, 92% of families approached in the ICU and asked to participate provided consent. By screening the MICU for 2 hours per day, 7 days per week, for up to 7 consecutive days, we were able to enroll a proxy for 38% of eligible patients. Patients with an enrolled proxy were more likely to be white, live in a zip code with a high median income, and had longer ICU stays and higher ICU mortality. Among eligible patients 15% had no visitors at any time during their ICU stay. Family availability and recruitment rates were similar on weekdays, evenings, and weekends, and family members enrolled after normal business hours had similar characteristics to those enrolled on weekdays.

As the effects of critical illness on ICU families[24,25,31] have been recognized, research aimed at understanding family-member needs[32–34], and improving their mental health outcomes[35,36] has expanded. Both eligibility criteria and consent rate for existing studies vary substantially. For example, some studies seek to enroll legal surrogate decision-makers[33], others try to identify the person who spends the most time caregiving for the patient[37], and many qualitative studies of ICU families use a convenience sample.[38] A recent review identified 14 randomized controlled trials of interventions designed to better meet the needs of ICU families, including face-to-face meetings, changes in visitation policies, and educational brochures.[39] To interpret the results of these trials it is important to understand how many ICU patients have family members who could potentially benefit from the intervention, and the representativeness of family members enrolled in the trials.

Requiring in-person recruitment and consent during a 2-hour daily enrollment period greatly limited our ability to enroll proxies for patients with short ICU stays as well as those families who visited infrequently or briefly. As a result, about half of eligible patients were not enrolled because a proxy was not available. This is comparable to previous evaluations of recruitment methods for ICU patients in which 40% – 80% of eligible patients who lacked decision-making capacity were not enrolled because no family member was available to provide surrogate consent.[40,41]

While the issue of critically ill, unbefriended patients (those without any family member or friend who can act as a surrogate decisions-maker) is well described in the medicolegal and bioethics literature,[42–44] the disadvantages of lacking someone who is regularly present at the patient’s bedside are less well documented. In addition to these patients and families being less likely to participate in research requiring in-person consent, these families may be less likely to benefit from multidisciplinary family meetings, participating in bedside rounds, communication facilitators[15], decision-aids requiring clinician supervision[14,45,46], and palliative care consultation [47]. Our data also suggest that the association between the absence of family members at the bedside and patient outcomes is likely confounded by socio-economic status and race.

Our high consent rate for approached families may be attributable, in part, to families being asked to complete a single low-burden interview generally requiring <30 minutes.[48] Although none of the study team members recruiting family members were physicians, all were trained to approach family members professionally and to allow families sufficient information, time, and space to make an informed decision about participation.[49] Recent qualitative research on the experiences of ICU families asked to participate in critical care research suggests that family members ultimately weigh the risk of participation against the potential benefit to the patient.[50,51] One of the “risks” associated with participation in our study was leaving the patient’s room and missing an update from a physician. We sought to mitigate this risk by explaining that interviews were conducted in a nearby room within the ICU and asking bedside nurses to interrupt the interview if a physician arrived. If families remained concerned, we offered to come back later in the 2-hour daily enrollment period or the next day.

Strengths of our study include its rigorous evaluation of a systematic and well-defined recruitment strategy to identify family members of ICU patients acting as patient proxies. However, the study also has potential limitations. For instance, the results of our study cannot be extrapolated to other more intensive recruitment strategies, or to other ICU populations such as pediatric or surgical ICUs. Moreover, our results may have also been substantially different if the study intervention required longitudinal follow-up, was interventional or invasive, or was directed at ICU patients without decision-making capacity. We recognize these limitations and encourage other investigators to conduct similar studies in their own institutions in preparation for performing larger scale trials.

In conclusion, a recruitment strategy involving limited human resources and time-varying, daily in-person screening over 7 days enrolled a family member for more than one third of eligible patients with 92% of approached family members agreeing to participate. However, patients with enrolled family members were disproportionately white, wealthy, mechanically ventilated, and had higher ICU mortality. Day of the week, and time of day were not associated with family presence in the ICU or consent rate. Investigators planning trials of interventions for ICU families should test recruitment plans to ensure their studies achieve appropriate power and generalizability.

## Supporting information

S1 Fig Schematic of ICU proxy recruitment strategy—Example using a hypothetical patient. (TIF)

S2 Fig Algorithm used to identify legal healthcare proxies. (PDF)

S3 Fig Availability of advance directives in patient charts and noted in electronic medical record. Abbreviations: AD, Advance directive. 1 Percentages do not sum to 100% due to rounding. Quoted language was asked of enrolled proxies by research assistants. Information within tables was obtained from the patient’s admission screening note in the electronic medical record.
(PNG)

## References

[1] Prendergast TJ, Claessens MT, Luce JM. A national survey of end-of-life care for critically ill patients. Am J Respir Crit Care Med. 1998;158: 1163–1167. 10.1164/ajrccm.158.4.9801108 9769276

[2] Torke AM, Sachs GA, Helft PR, Montz K, Hui SL, Slaven JE, et al Scope and outcomes of surrogate decision making among hospitalized older adults. JAMA Intern Med. 2014;174: 370–377. 10.1001/jamainternmed.2013.13315 24445375 PMC3947481

[3] Davidson JE, Powers K, Hedayat KM, Tieszen M, Kon AA, Shepard E, et al Clinical practice guidelines for support of the family in the patient-centered intensive care unit: American College of Critical Care Medicine Task Force 2004–2005. Crit Care Med. 2007;35: 605–622. 10.1097/01.CCM.0000254067.14607.EB 17205007

[4] Kon AA, Davidson JE, Morrison W, Danis M, White DB. Shared Decision Making in ICUs: An American College of Critical Care Medicine and American Thoracic Society Policy Statement. Crit Care Med. 2015; 10.1097/CCM.0000000000001396 26509317 PMC4788386

[5] Nelson JE, Puntillo KA, Pronovost PJ, Walker AS, McAdam JL, Ilaoa D, et al In their own words: patients and families define high-quality palliative care in the intensive care unit. Crit Care Med. 2010;38: 808–818. 20198726 10.1097/ccm.0b013e3181c5887c PMC3267550

[6] Schenker Y, Crowley-Matoka M, Dohan D, Tiver GA, Arnold RM, White DB. I don’t want to be the one saying “we should just let him die”: intrapersonal tensions experienced by surrogate decision makers in the ICU. J Gen Intern Med. 2012;27: 1657–1665. 10.1007/s11606-012-2129-y 23011253 PMC3509291

[7] Nunez ER, Schenker Y, Joel ID, Reynolds CF, Dew MA, Arnold RM, et al Acutely Bereaved Surrogates’ Stories About the Decision to Limit Life Support in the ICU. Crit Care Med. 2015; 10.1097/CCM.0000000000001270 26327201 PMC4607641

[8] Abbott KH, Sago JG, Breen CM, Abernethy AP, Tulsky JA. Families looking back: one year after discussion of withdrawal or withholding of life-sustaining support. Crit Care Med. 2001;29: 197–201. 11176185 10.1097/00003246-200101000-00040

[9] Schuster RA, Hong SY, Arnold RM, White DB. Investigating conflict in ICUs-is the clinicians’ perspective enough? Crit Care Med. 2014;42: 328–335. 10.1097/CCM.0b013e3182a27598 24434440 PMC3902111

[10] Chiarchiaro J, Schuster RA, Ernecoff NC, Barnato AE, Arnold RM, White DB. Developing a Simulation to Study Conflict in Intensive Care Units. Ann Am Thorac Soc. 2015;12: 526–532. 10.1513/AnnalsATS.201411-495OC 10.1513/AnnalsATS.201411-495OC 25643166 PMC4418329

[11] LeClaire MM, Oakes JM, Weinert CR. Communication of prognostic information for critically ill patients. Chest. 2005;128: 1728–1735. 10.1378/chest.128.3.1728 16162781

[12] Lee Char SJ, Evans LR, Malvar GL, White DB. A randomized trial of two methods to disclose prognosis to surrogate decision makers in intensive care units. Am J Respir Crit Care Med. 2010;182: 905–909. 10.1164/rccm.201002-0262OC 20538959 PMC2970862

[13] Chiarchiaro J, Buddadhumaruk P, Arnold RM, White DB. Quality of communication in the ICU and surrogate’s understanding of prognosis. Crit Care Med. 2015;43: 542–548. 10.1097/CCM.0000000000000719 25687030 PMC4336600

[14] Cox CE, Lewis CL, Hanson LC, Hough CL, Kahn JM, White DB, et al Development and pilot testing of a decision aid for surrogates of patients with prolonged mechanical ventilation. Crit Care Med. 2012;40: 2327–2334. 10.1097/CCM.0b013e3182536a63 22635048 PMC3826165

[15] Curtis JR, Treece PD, Nielsen EL, Gold J, Ciechanowski PS, Shannon SE, et al Randomized Trial of Communication Facilitators to Reduce Family Distress and Intensity of End-of-Life Care. Am J Respir Crit Care Med. 2015;193: 154–162. 10.1164/rccm.201505-0900OC 26378963 PMC4731711

[16] Van Pelt DC, Milbrandt EB, Qin L, Weissfeld LA, Rotondi AJ, Schulz R, et al Informal Caregiver Burden among Survivors of Prolonged Mechanical Ventilation. Am J Respir Crit Care Med. 2007;175: 167–173. 10.1164/rccm.200604-493OC 17068327 PMC1899280

[17] Douglas SL, Daly BJ, O’Toole E, Hickman RL Jr.. Depression among white and nonwhite caregivers of the chronically critically ill. J Crit Care. 2010;25: 364.e11–364.e19. 10.1016/j.jcrc.2009.09.004 19836923 PMC2883690

[18] Choi J, Sherwood PR, Schulz R, Ren D, Donahoe MP, Given B, et al Patterns of depressive symptoms in caregivers of mechanically ventilated critically ill adults from intensive care unit admission to 2 months postintensive care unit discharge: A pilot study. Crit Care Med. 2012; 10.1097/CCM.0b013e3182451c58 22430242 PMC3330166

[19] Hwang DY, Yagoda D, Perrey HM, Currier PF, Tehan TM, Guanci M, et al Anxiety and depression symptoms among families of adult intensive care unit survivors immediately following brief length of stay. J Crit Care. 10.1016/j.jcrc.2013.11.022 24411107

[20] Jones C, Skirrow P, Griffiths RD, Humphris G, Ingleby S, Eddleston J, et al Post-traumatic stress disorder-related symptoms in relatives of patients following intensive care. Intensive Care Med. 2004;30: 456–460. 10.1007/s00134-003-2149-5 14767589

[21] Azoulay E, Pochard F, Kentish-Barnes N, Chevret S, Aboab J, Adrie C, et al Risk of post-traumatic stress symptoms in family members of intensive care unit patients. Am J Respir Crit Care Med. 2005;171: 987–994. 10.1164/rccm.200409-1295OC 15665319

[22] Anderson WG, Arnold RM, Angus DC, Bryce CL. Posttraumatic stress and complicated grief in family members of patients in the intensive care unit. J Gen Intern Med. 2008;23: 1871–1876. 10.1007/s11606-008-0770-2 18780129 PMC2585673

[23] McAdam JL, Fontaine DK, White DB, Dracup KA, Puntillo KA. Psychological symptoms of family members of high-risk intensive care unit patients. Am J Crit Care Off Publ Am Assoc Crit-Care Nurses. 2012;21: 386–393; quiz 394. 10.4037/ajcc2012582 23117902

[24] Needham DM, Davidson J, Cohen H, Hopkins RO, Weinert C, Wunsch H, et al Improving long-term outcomes after discharge from intensive care unit: report from a stakeholders’ conference. Crit Care Med. 2012;40: 502–509. 10.1097/CCM.0b013e318232da75 21946660

[25] Davidson JE. Time for a formal assessment, treatment, and referral structure for families of intensive care unit patients. Crit Care Med. 2012;40: 1675–1676. 10.1097/CCM.0b013e318249594a 22511159

[26] Downar J, Barua R, Sinuff T. The desirability of an Intensive Care Unit (ICU) Clinician-Led Bereavement Screening and Support Program for Family Members of ICU Decedents (ICU Bereave). J Crit Care Phila. 2014;29: 311.e9–16. 10.1016/j.jcrc.2013.11.024 24581934

[27] Hernán MA, Hernández-Díaz S, Robins JM. A structural approach to selection bias. Epidemiology. 2004;15: 615–625. 15308962 10.1097/01.ede.0000135174.63482.43

[28] Davidson JE. Neglect of quality-of-life considerations in intensive care unit family meetings for long-stay intensive care unit patients. Crit Care Med. 2012;40: 671–672. 10.1097/CCM.0b013e3182372998 22249044

[29] Schwartz AAGJS. Who Can Make Health Care Decisions For Another? Defining Health Care Proxies Under Maryland Law [Internet]. 2006. http://www.marylandattorneygeneral.gov/Health%20Policy%20Documents/proxies_definition.pdf

[30] Frosh, Attorney General Brian E. Summary Of Maryland Health Care Decisions Act [Internet]. 2014. http://www.marylandattorneygeneral.gov/Health%20Policy%20Documents/HCDAsummary.pdf

[31] Netzer G, Sullivan DR. Recognizing, naming, and measuring a family intensive care unit syndrome. Ann Am Thorac Soc. 2014;11: 435–441. 10.1513/AnnalsATS.201309-308OT 24673699 PMC4028736

[32] Kentish-Barnes N, Lemiale V, Chaize M, Pochard F, Azoulay E. Assessing burden in families of critical care patients. Crit Care Med. 2009;37: S448–456. 10.1097/CCM.0b013e3181b6e145 20046134

[33] Lemiale V, Kentish-Barnes N, Chaize M, Aboab J, Adrie C, Annane D, et al Health-Related Quality of Life in Family Members of Intensive Care Unit Patients. J Palliat Med. 2010;13: 1131–1137. 10.1089/jpm.2010.0109 20836638

[34] Anderson WG, Cimino JW, Ernecoff NC, Ungar A, Shotsberger KJ, Pollice LA, et al A Multicenter Study of Key Stakeholders’ Perspectives on Communicating with Surrogates about Prognosis in Intensive Care Units. Ann Am Thorac Soc. 2014;12: 142–152. 10.1513/AnnalsATS.201407-325OC 25521191 PMC4342839

[35] Curtis JR, Treece PD, Nielsen EL, Gold J, Ciechanowski PS, Shannon SE, et al Randomized Trial of Communication Facilitators to Reduce Family Distress and Intensity of End-of-life Care. Am J Respir Crit Care Med. 2015; 10.1164/rccm.201505-0900OC 26378963 PMC4731711

[36] Mistraletti G, Umbrello M, Mantovani ES, Moroni B, Formenti P, Spanu P, et al A family information brochure and dedicated website to improve the ICU experience for patients’ relatives: an Italian multicenter before-and-after study. Intensive Care Med. 2016; 1–11. 10.1007/s00134-016-4592-0 27830281

[37] Douglas SL, Daly BJ, Kelley CG, O’Toole E, Montenegro H. Impact of a Disease Management Program Upon Caregivers of Chronically Critically Ill Patients. Chest. 2005;128: 3925–3936. 10.1378/chest.128.6.3925 16354865

[38] Ernecoff NC, Witteman HO, Chon K, Chen YI, Buddadhumaruk P, Chiarchiaro J, et al Key stakeholders’ perceptions of the acceptability and usefulness of a tablet-based tool to improve communication and shared decision making in ICUs. J Crit Care. 2016;33: 19–25. 10.1016/j.jcrc.2016.01.030 27037049

[39] Kynoch K, Chang A, Coyer F, McArdle A. The effectiveness of interventions to meet family needs of critically ill patients in an adult intensive care unit: a systematic review update. JBI Database Syst Rev Implement Rep. 2016;14: 181–234. 10.11124/JBISRIR-2016-2477 27532144

[40] Grap MJ, Munro CL. Subject recruitment in critical care nursing research: a complex task in a complex environment. Heart Lung J Crit Care. 2003;32: 162–168. 10.1016/s0147-9563(03)00031-1 12827101

[41] Larkin ME, Beauharnais CC, Magyar K, Macey L, Grennan KB, Boykin EE, et al Obtaining surrogate consent for a minimal-risk research study in the intensive care unit setting. Clin Trials. 2013;10: 93–96. 10.1177/1740774512464727 23169873

[42] White DB, Curtis JR, Wolf LE, Prendergast TJ, Taichman DB, Kuniyoshi G, et al Life support for patients without a surrogate decision maker: who decides? Ann Intern Med. 2007;147: 34–40. 17606959 10.7326/0003-4819-147-1-200707030-00006

[43] Marks MAZ, Arkes HR. Patient and surrogate disagreement in end-of-life decisions: can surrogates accurately predict patients’ preferences? Med Decis Mak Int J Soc Med Decis Mak. 2008;28: 524–531. 10.1177/0272989X08315244 18566485

[44] Cohen AB, Wright MS, Cooney L Jr, Fried T. Guardianship and end-of-life decision making. JAMA Intern Med. 2015; 10.1001/jamainternmed.2015.3956 26258634 PMC4683611

[45] Volandes AE, Mitchell SL, Gillick MR, Chang Y, Paasche-Orlow MK. Using video images to improve the accuracy of surrogate decision-making: a randomized controlled trial. J Am Med Dir Assoc. 2009;10: 575–580. 10.1016/j.jamda.2009.05.006 19808156 PMC2782701

[46] Cox CE, Wysham NG, Walton B, Jones D, Cass B, Tobin M, et al Development and usability testing of a Web-based decision aid for families of patients receiving prolonged mechanical ventilation. Ann Intensive Care. 2015;5: 6 10.1186/s13613-015-0045-0 25852965 PMC4385299

[47] Sudore RL, Casarett D, Smith D, Richardson DM, Ersek M. Family involvement at the end-of-life and receipt of quality care. J Pain Symptom Manage. 2014;48: 1108–1116. 10.1016/j.jpainsymman.2014.04.001 24793077

[48] Kross EK, Nielsen EL, Curtis JR, Engelberg RA. Survey burden for family members surveyed about end-of-life care in the intensive care unit. J Pain Symptom Manage. 2012;44: 671–680. 10.1016/j.jpainsymman.2011.11.008 22762964 PMC3488148

[49] Burns KEA, Rizvi L, Smith OM, Lee Y, Lee J, Wang M, et al Is there a role for physician involvement in introducing research to surrogate decision makers in the intensive care unit? (The Approach trial: a pilot mixed methods study). Intensive Care Med. 2014;41: 58–67. 10.1007/s00134-014-3558-3 25491659

[50] Burns KEA, Zubrinich C, Tan W, Raptis S, Xiong W, Smith O, et al Research Recruitment Practices and Critically Ill Patients. Am J Respir Crit Care Med. 2013;187: 1212–1218. 10.1164/rccm.201208-1537OC 23525935

[51] Burns KEA, Prats CJ, Maione M, Lanceta M, Zubrinich C, Jeffs L, et al The Experience of Surrogate Decision Makers on Being Approached for Consent for Patient Participation in Research. A Multicenter Study. Ann Am Thorac Soc. 2016;14: 238–245. 27849142 10.1513/AnnalsATS.201606-425OC

