# Supplementary figures and images for: Evaluation of a strategy for enrolling the families of critically ill patients in research using limited human resources
Source: PLoS One. 2017 May 25;12(5):e0177741. doi: 10.1371/journal.pone.0177741 (PMC5444627; doi:10.1371/journal.pone.0177741)

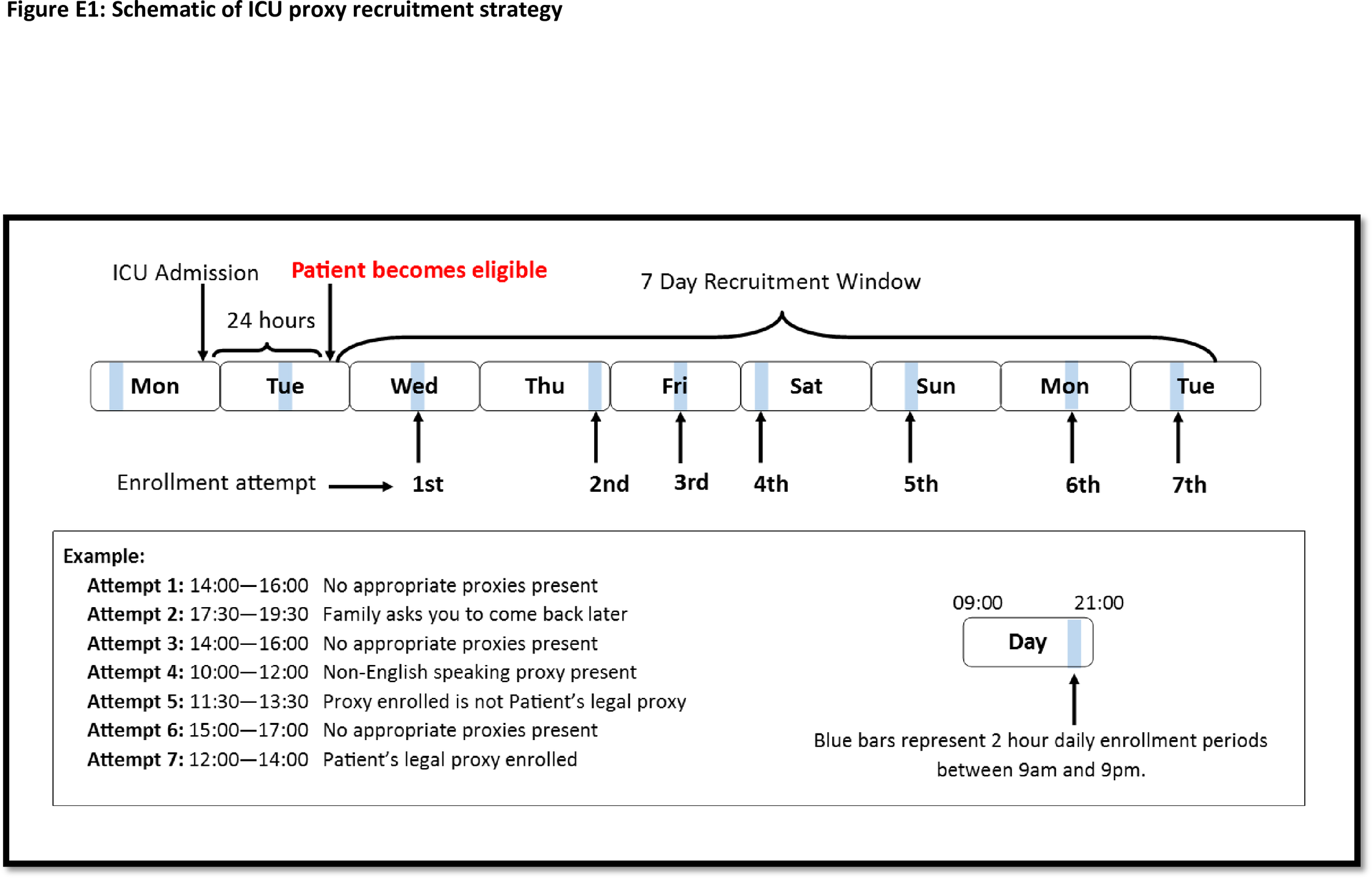

Supplement: S1 Fig — (TIF) [file pone.0177741.s001.tif]

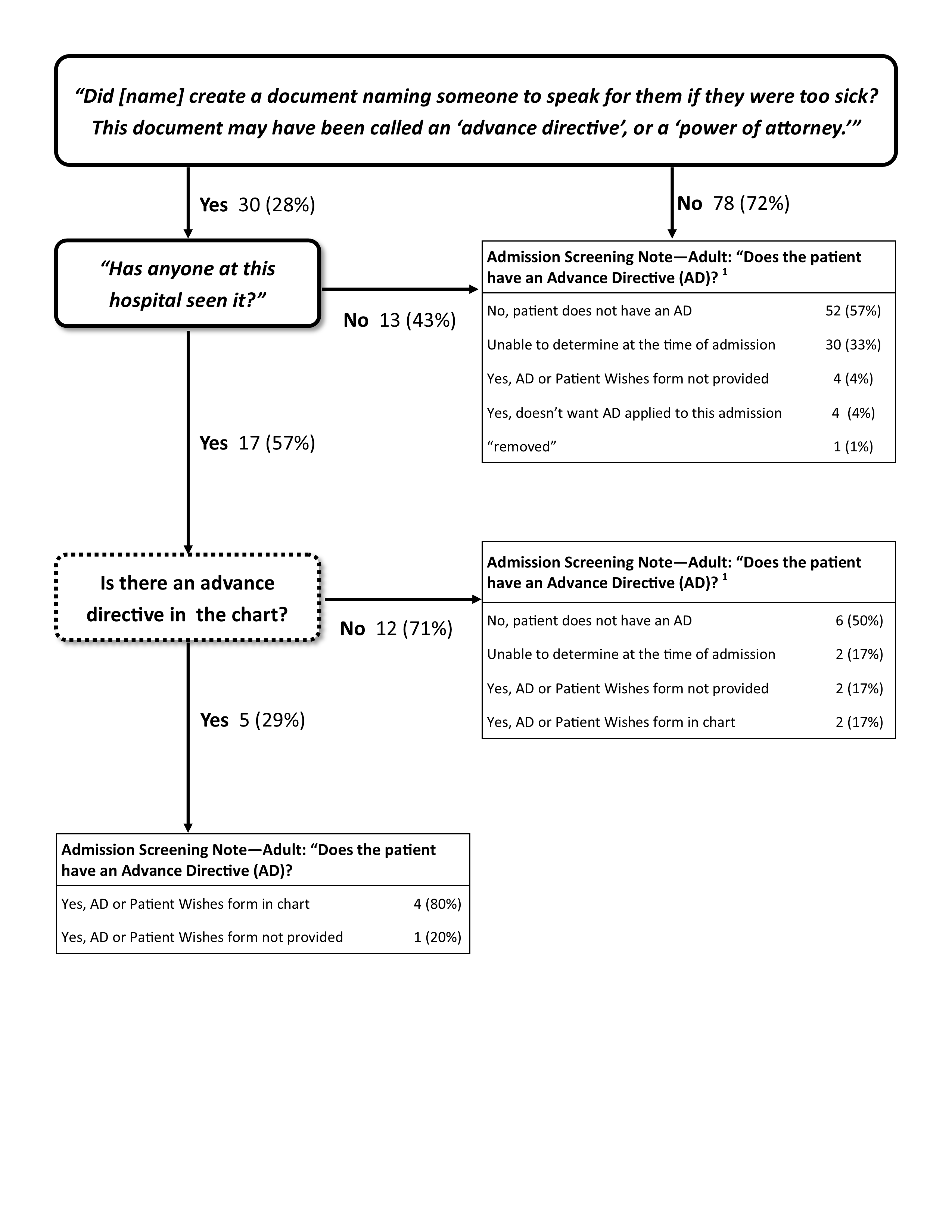

Supplement: S3 Fig — Abbreviations: AD, Advance directive. 1 Percentages do not sum to 100% due to rounding. Quoted language was asked of enrolled proxies by research assistants. Information within tables was obtained from the patient’s admission screening note in the electronic medical record. (PNG) [file pone.0177741.s003.png]
