# Supplementary material for: Evaluation of a strategy for enrolling the families of critically ill patients in research using limited human resources
Source: PLoS One. 2017 May 25;12(5):e0177741. doi: 10.1371/journal.pone.0177741 (PMC5444627; doi:10.1371/journal.pone.0177741)
Supplement: S2 Fig — (PDF) [file pone.0177741.s002.pdf]

PAB-Pilot Form 2: Family Interview

Instructions: Circle all “yes” or “no” answers. Only speak aloud the text in yellow boxes.

Subject ID - 1:______________
Data collector initials:_______
Date:__________________

“Did [name] create a document naming someone to speak for them if they are too sick to make decisions? This document may have been called an ***advance directive****, or a* ***power of attorney****.”*

NO → Ask questions below. If >1 person qualifies in 1 box, record the name of the person who has spent the most time talking to ICU providers.

YES → *“Where is that document now? Has anyone at this hospital seen it?”*

- NO → *“Did [name] tell an ICU doctor who should speak for him/her?”*
  - NO → Ask questions below.
  - YES → **Say:** *“Who did [name] say should speak for him/her?“*
    Name:_________________
    Relations: _____________
- YES → Is the document recorded in the patient’s chart?
  - NO → If a document naming a heath care agent was NOT identified in the patient’s medical record (Form 1, question F), do **NOT** contradict the subject. (See after interview box below) → **Say:** *“Who did [name] say should speak for him/her?“*
  - YES → If a document IS in the chart, the person named in the document is a health care agent and **the legal proxy**. Document does not need to be reviewed for proper witnesses. → *“According to that document, who may speak for [name]?”* → STOP

Say: *“Is [name] married?”* — YES → STOP; NO ↓

“Does [name] live with a boyfriend, girlfriend, or partner? Do they share expenses such as splitting rent or co-owning property?” — YES → STOP; NO ↓

“Does [name] have any children who are at least 18 years old?” — YES → STOP; NO ↓

“Are either of [name’s] parents alive?” — YES → STOP; NO ↓

“Does [name] have brothers and sisters who are at least 18 years old?” — YES → STOP; NO ↓

“Who in the world is closest to [name]? Which friend or family member sees [name] regularly or may have known about [name’s] health and personal beliefs? “ — YES → STOP

STOP: This is the legal proxy
Name: _____________
Relation: ___________

After interview

Use the results of this flowchart to complete questions 9—12 of Form 3: Family Interview.

If subject believes the hospital possess a copy of a legal document naming a health care agent, but you can’t find that document in the patient’s medical record, please alert a provider.

This algorithm was designed for use in the PAB-pilot study (IRB_00080137). Hospitalized prisoners and wards of the state are not eligible to participate in this study and thus do not appear in the algorithm.
